# Cat Bite-Associated Infections of the Upper Extremity Requiring Surgical Treatment: A Report of Two Cases Involving Pasteurella multocida and Capnocytophaga felis

**DOI:** 10.7759/cureus.105237

**Published:** 2026-03-14

**Authors:** Kosuke Saito, Ryo Hosomi, Toshiki Minato, Hiroki Kishi, Masanori Matsuura

**Affiliations:** 1 Department of Orthopedic Surgery, Osaka City General Hospital, Osaka City, JPN

**Keywords:** animal bite, capnocytophaga spp, cat bite, genetic test, osteomyelitis, pasteurella multocida, upper extremities

## Abstract

Although cat bite-associated infections of the upper extremity are common, their impact might be underestimated due to the mild initial clinical findings. However, such infections could progress to deep musculoskeletal involvement and require surgical intervention. Herein, we report two cases of cat bite-associated infections of the upper extremities that required surgical treatment. The first case involved a rapidly progressing forearm infection caused by *Pasteurella multocida* that was successfully managed with surgical debridement and appropriate antimicrobial therapy. In contrast, the second case comprised an indolent clinical course with osteomyelitis of the first metacarpal bone and minimal inflammatory findings. Conventional cultures failed to identify the causative organism, and genetic testing was required to detect *Capnocytophaga felis*. The patient was successfully treated with surgical debridement and prolonged antimicrobial therapy, with no sign of recurrence. To the best of our knowledge, this is the first report of osteomyelitis caused by this organism. These contrasting clinical courses underscore the diversity of the feline oral microbiota and the causative pathogen-dependent disease presentation variability. When clinical improvement is not achieved with initial medical management, timely surgical intervention is essential. Furthermore, when the causative pathogen cannot be identified using conventional culture methods, genetic testing should be considered to ensure accurate diagnosis and appropriate treatment.

## Introduction

Cat bites are a common cause of animal bite injuries and account for approximately 20%-30% of all bite-related presentations [1]. A previous study reported that 20%-80% of cat bite wounds may become infected [2]. Although many cat bite-associated infections can be managed conservatively, delayed recognition or inadequate initial assessment may result in deep musculoskeletal infections requiring surgical intervention [3]. Cat oral microbiota is diverse, with a clinical course that could vary based on the causative pathogen. While *Pasteurella multocida* is the most frequently isolated organism, other pathogens, including *Capnocytophaga* species, have increasingly been recognized as clinically relevant [4,5]. In some cases, culture-negative infections present significant diagnostic challenges, and molecular identification techniques may be required to establish an etiological diagnosis when conventional culture methods fail. In this report, we present two cases of cat bite-associated upper extremity infections caused by *Pasteurella multocida* and *Capnocytophaga felis*, respectively, both of which required surgical treatment after the failure of initial empirical antimicrobial therapy. The *Pasteurella multocida* case exhibited a rapidly progressive clinical course with evident inflammatory findings, whereas the *Capnocytophaga felis* case followed an indolent course with minimal inflammatory response and negative conventional cultures, requiring polymerase chain reaction (PCR) sequencing for pathogen identification. These cases highlight the variability in clinical course depending on the causative pathogen and underscore the importance of appropriate diagnostic strategies, including molecular identification in culture-negative infections. To the best of our knowledge, this study describes the first *Capnocytophaga felis*-induced osteomyelitis.

## Case presentation

Case 1

**Figure 1:**
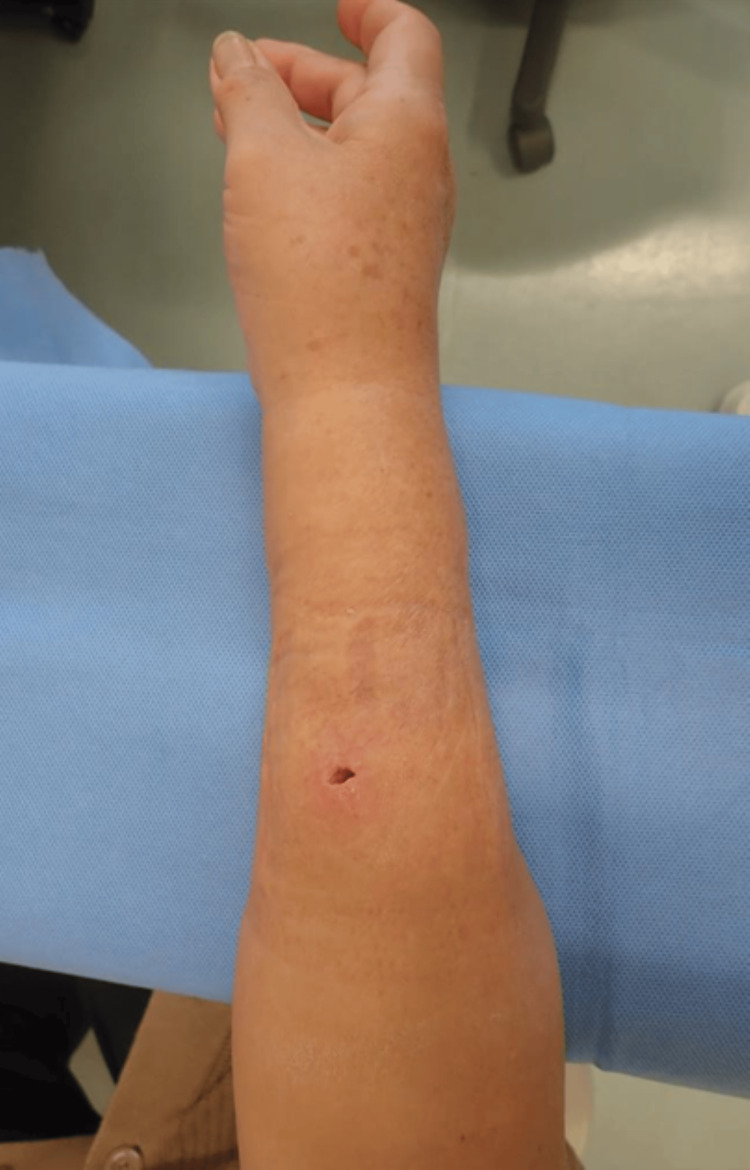
Clinical photograph The clinical photograph of the right forearm in Case 1 reveals a fistula resulting from the spontaneous rupture of the abscess

A 63-year-old woman sustained a bite injury on her right forearm caused by a domestic cat. The patient developed erythema and local warmth the day after the injury. She initially presented to a local dermatology clinic, where oral cephalosporin was prescribed. However, her symptoms worsened, and she visited a local orthopedic clinic seven days after the injury. At the time of evaluation at the second clinic, the abscess had spontaneously ruptured through a sinus tract, resulting in purulent discharge (Figure 1). Wound extension, irrigation, the placement of gauze packing, and the submission of wound cultures were performed, and intravenous piperacillin sodium was administered at the second clinic. The patient visited the second institution several times; however, her symptoms did not improve. Given the need for definitive source control measures, the patient was referred to our institution 12 days after the injury.

**Figure 2:**
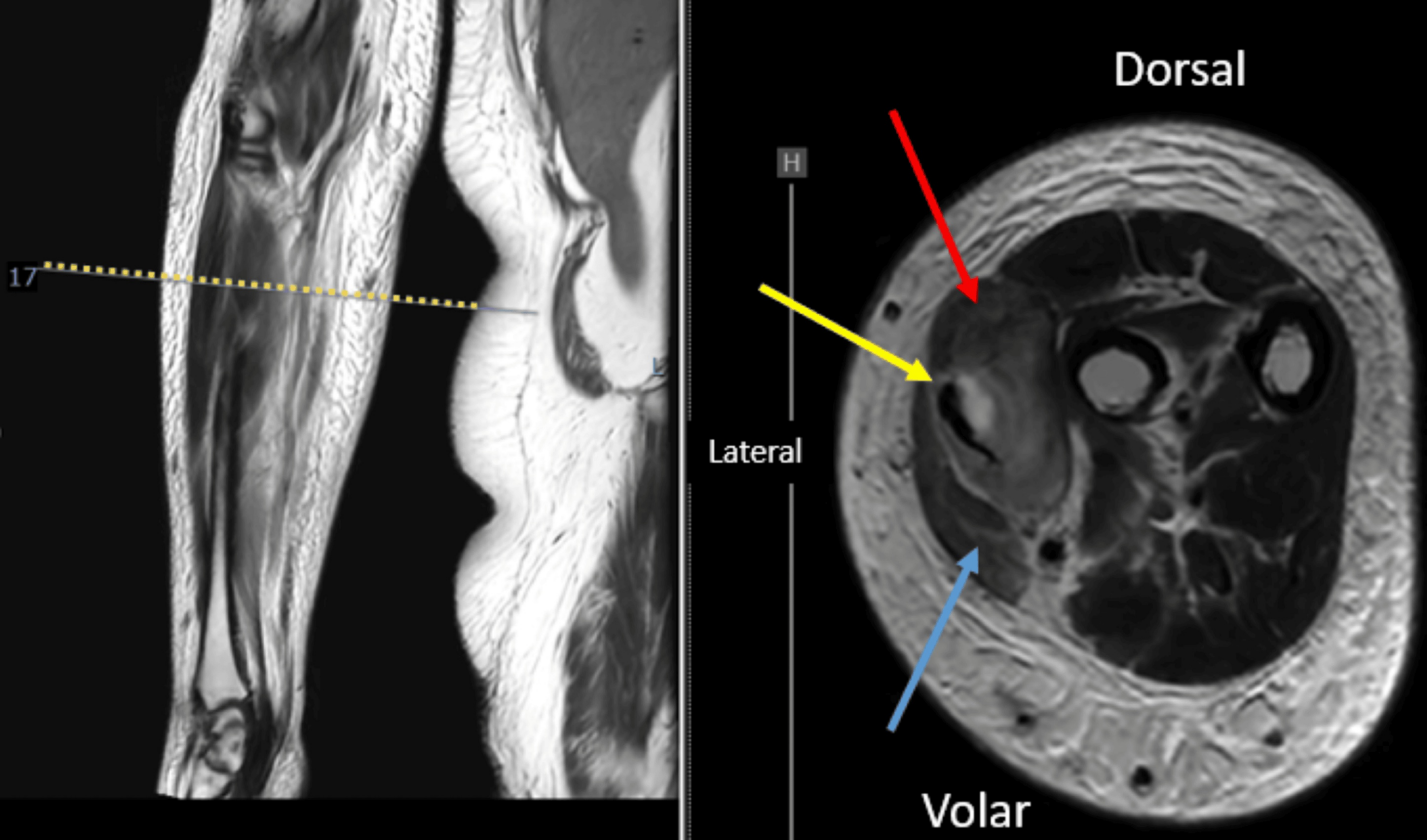
Magnetic resonance imaging The magnetic resonance imaging of the right forearm in Case 1 reveals an abscess within the lateral compartment. Red arrow, extensor carpi radialis muscles; blue arrow, brachioradialis muscle; yellow arrow, the abscess cavity

**Figure 3:**
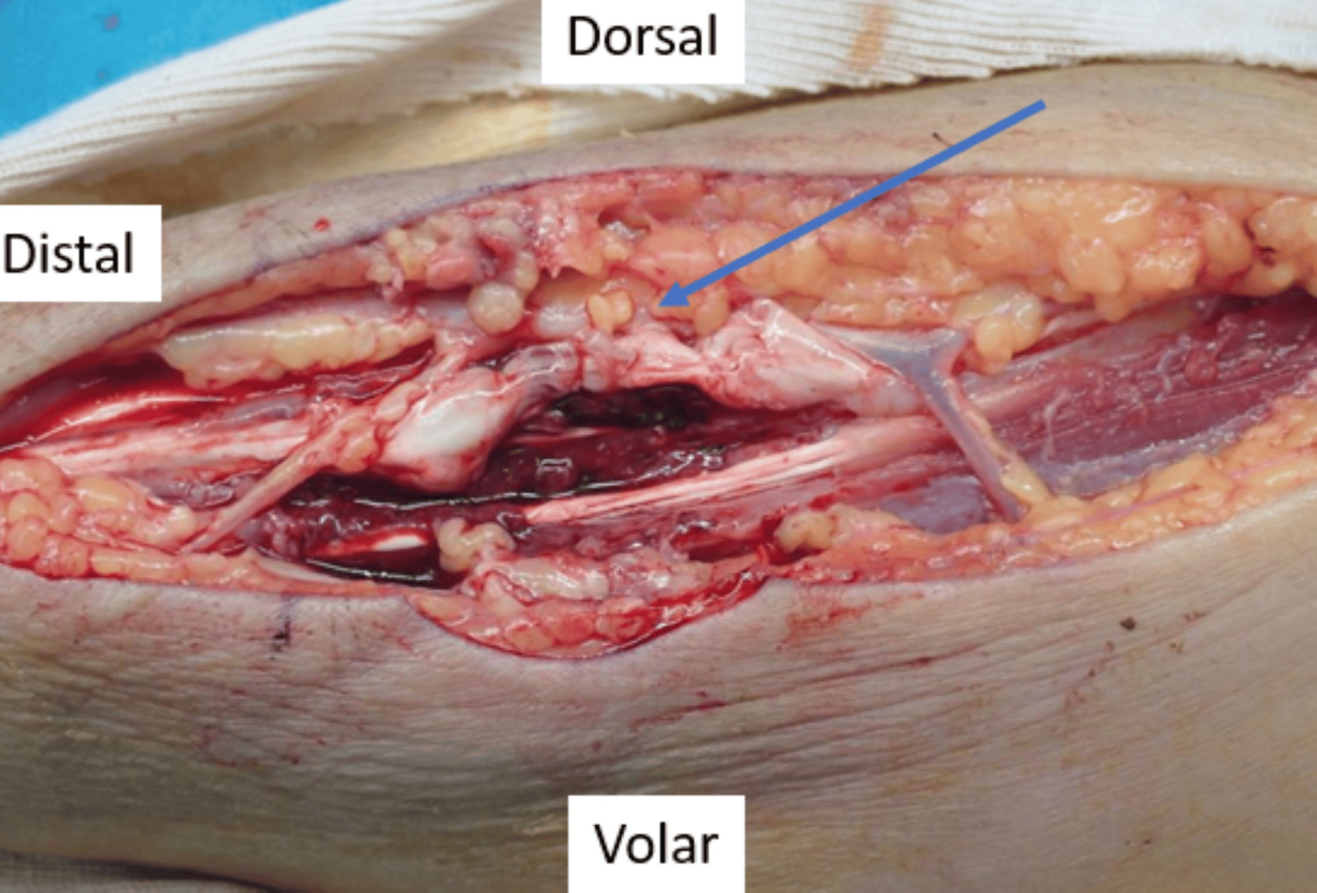
Intraoperative findings in Case 1 The blue arrow indicates the partial necrosis and degeneration of the extensor carpi radialis longus tendon without complete rupture

**Figure 4:**
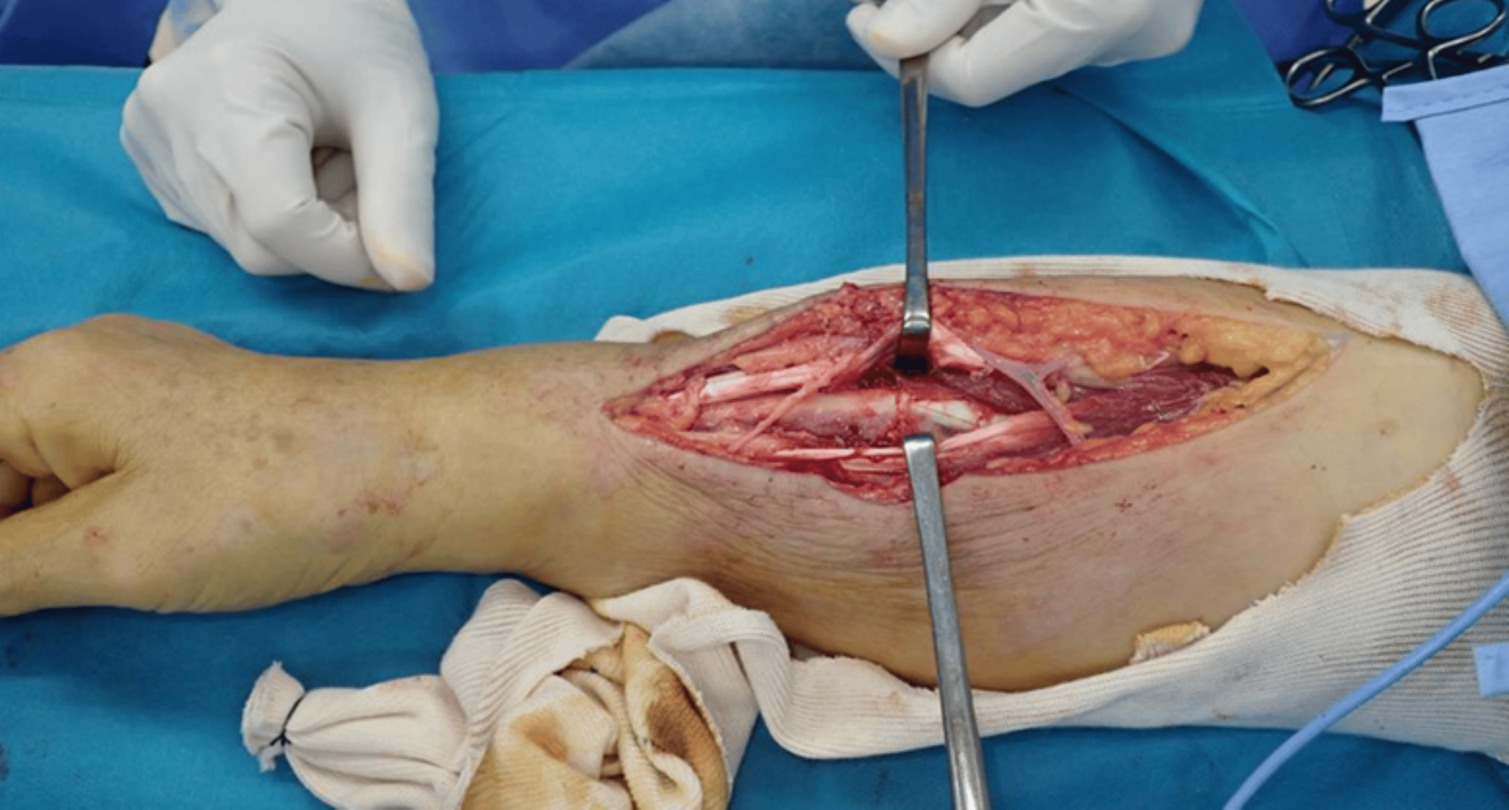
Postoperative photograph of Case 1 The extensive surgical debridement of necrotic tissue within the lateral forearm compartment was performed

In magnetic resonance imaging, T2-weighted imaging revealed an abscess in the right forearm (Figure 2). Laboratory investigations revealed a white blood cell count of 10,130/µL and a C-reactive protein level of 1.8 mg/dL, indicating a mild to moderate inflammatory response (Table 1). The patient had a history of diabetes mellitus. Definitive intervention was performed 14 days after the injury. Surgical exploration was performed through a 15 cm longitudinal incision along the lateral compartment of the forearm. Upon exposure, white, purulent material was expelled from the sinus tract. The brachioradialis and extensor carpi radialis longus tendons were partially necrotic and degenerating owing to the infection; however, complete tendon rupture was not observed (Figure 3). Extensive surgical debridement was performed, including the removal of necrotic tissue, involving the muscle and tendons (Figure 4). The wound was thoroughly irrigated with saline solution. The fascia was left open, a Penrose drain was placed, and the wound was closed. The drain was removed on postoperative day 6 after confirming improvement in the local infection. Postoperatively, the affected limb was immobilized with a long-arm cast for two weeks. Wound cultures revealed the presence of *Pasteurella multocida*. The patient was treated with intravenous ampicillin/sulbactam for one week and then transitioned to oral amoxicillin/clavulanate. Antibiotic therapy was discontinued three weeks after surgery. The duration of antimicrobial therapy was determined in consultation with the infection control team at our institution, considering the patient’s clinical response. No infection recurrence was observed during follow-up.

**Table 1:** Laboratory values (Case 1)

Laboratory tests (units)	Result	Reference range
Hemoglobin (g/dL)	13.4	11.6-14.8
White blood cells (/µL)	10,130	3,300-8,600
Platelets (K/µL)	452	158-348
Sodium (mEq/L)	143	138-145
Potassium (mEq/L)	4.3	3.6-4.8
Chloride (mEq/L)	109	101-108
C-reactive protein (mg/dL)	1.8	<0.30

Case 2

**Figure 5:**
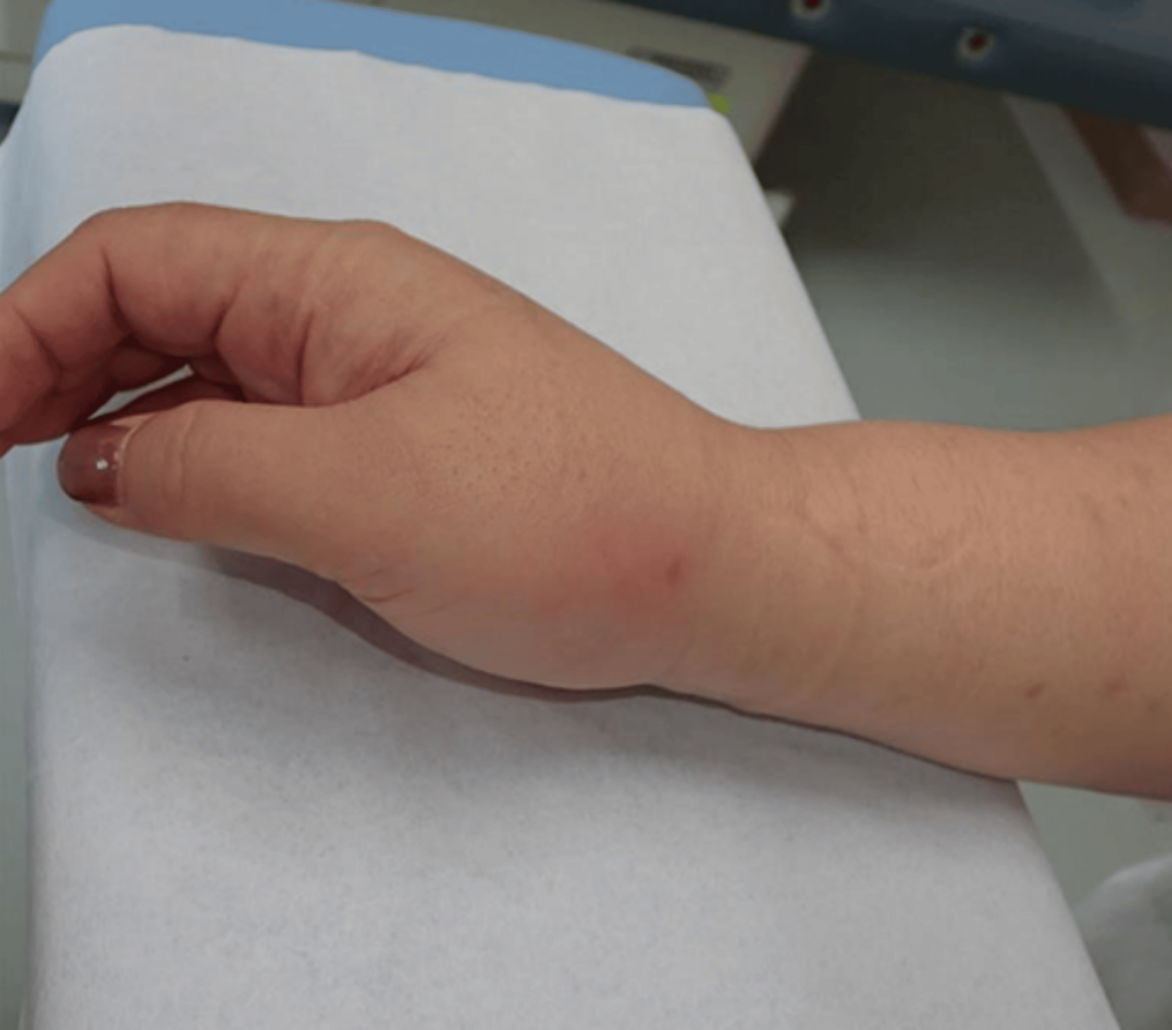
Clinical appearance of the right thumb on initial presentation for Case 2 A bite-wound scar is observed at the base of the thumb with surrounding erythema and swelling

**Figure 6:**
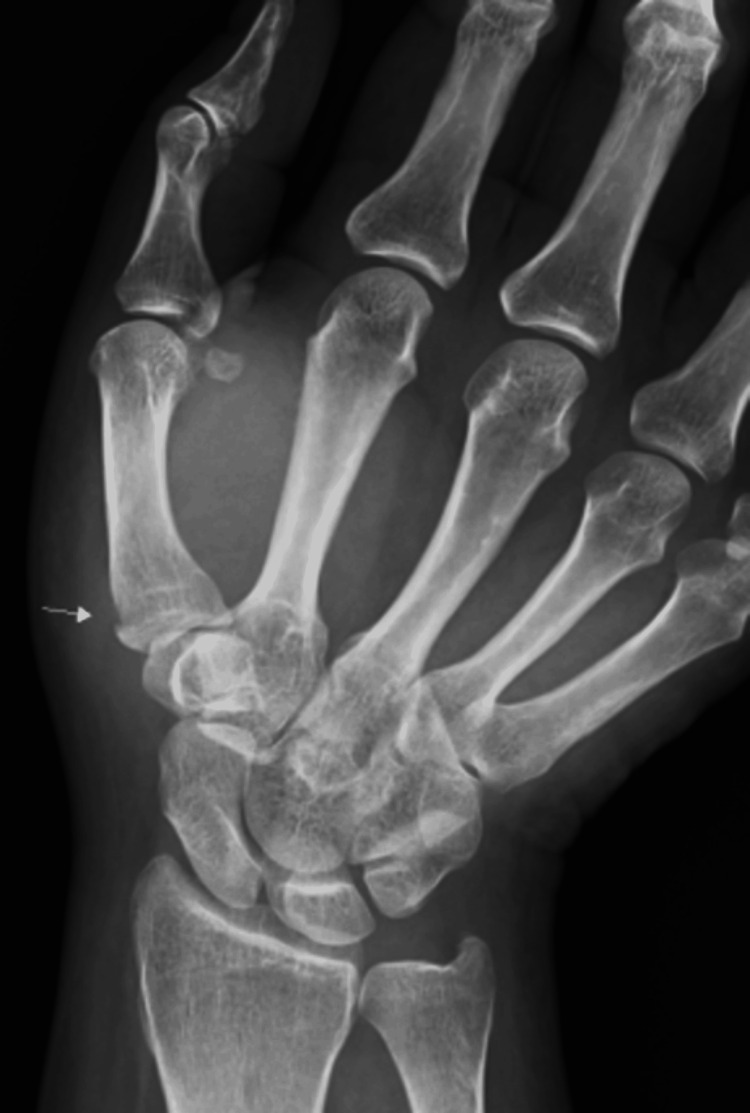
Plain radiograph of the right hand in Case 2 The white arrow indicates osteolytic changes at the base of the first metacarpal bone

**Figure 7:**
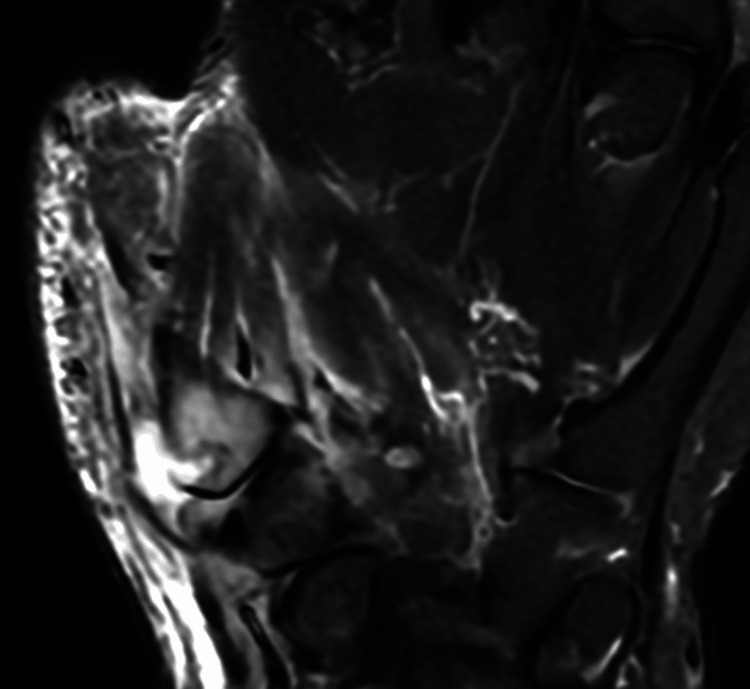
Magnetic resonance imaging T2-weighted magnetic resonance image of the right hand in Case 2 showing inflammatory changes extending into the bone marrow of the first metacarpal bone

A 49-year-old woman sustained a bite injury to her right thumb caused by her domestic cat. The patient initially received oral fluoroquinolone antibiotics. Within several days, the prominent symptoms ameliorated; although the mild pain persisted, the treatment was discontinued based on the decision of the patient and her attending physician. Subsequently, the patient was incidentally evaluated at a local orthopedic clinic after an unrelated traffic accident. During this visit, plain radiographs obtained for evaluation revealed osteolytic changes at the base of the right first metacarpal bone. Approximately five weeks after the cat bite injury, she was referred to our hospital for further assessment. Physical examination at presentation revealed a small bite-wound scar at the base of the right thumb, accompanied by surrounding erythema and swelling (Figure 5). Laboratory investigations revealed a white blood cell count of 5,400/µL and a C-reactive protein level of 0.09 mg/dL, both within normal limits (Table 2). Plain radiography confirmed osteolysis at the base of the right first metacarpal (Figure 6). In magnetic resonance imaging, T2-weighted imaging revealed inflammatory changes extending along the radial aspect of the first metacarpal with continuity into the bone marrow (Figure 7). These findings were consistent with abscess formation secondary to osteomyelitis.

**Table 2:** Laboratory values (Case 2)

Laboratory tests (units)	Result	Reference range
Hemoglobin (g/dL)	12.8	11.6-14.8
White blood cells (/µL)	5,400	3,300-8,600
Platelets (K/µL)	190	158-348
Sodium (mEq/L)	142	138-145
Potassium (mEq/L)	4.3	3.6-4.8
Chloride (mEq/L)	106	101-108
C-reactive protein (mg/dL)	0.1	<0.30

**Figure 8:**
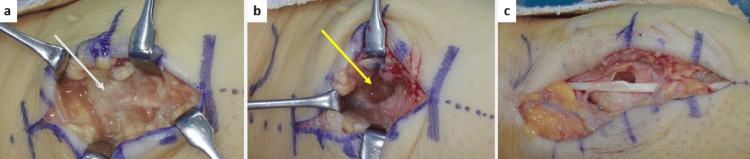
Intraoperative findings in Case 2 (A) Necrotic tissue around the first metacarpal bone was observed. White arrow: the abscess cavity. (B) An osteolytic defect with cortical perforation was observed in the first metacarpal bone. Yellow arrow: the cortical perforation. (C) Curettage of the medullary cavity through an osteolytic cortical defect

Surgical intervention was performed approximately six weeks after the injury. A surgical incision was made over the thumb carpometacarpal joint, and the approach was performed between the extensor pollicis brevis and extensor pollicis longus tendons. The superficial branch of the radial nerve was identified and preserved. The necrotic tissue caused by infection was extensively debrided (Figure 8A). An osteolytic defect with cortical perforation was observed in the first metacarpal bone (Figure 8B), and the curettage of the medullary cavity was performed (Figure 8C). The gross involvement of the carpometacarpal joint was not observed. The wound was thoroughly irrigated with copious amounts of saline, a Penrose drain was placed, and the skin was closed. The drain was removed on postoperative day 6 after confirming improvement in the local infection. Postoperatively, the patient was administered intravenous ampicillin/sulbactam for two weeks. Conventional wound cultures failed to identify the causative pathogen; therefore, genetic testing was performed. DNA was extracted from intraoperative tissue specimens. Broad-range bacterial *16S rRNA* gene PCR was performed targeting conserved regions of the *16S rRNA* gene. Sequence alignment using the National Center for Biotechnology Information (NCBI) Basic Local Alignment Search Tool (BLAST) (Bethesda, MD) database demonstrated 100% identity (463/463 base pairs {bp}) with *Capnocytophaga felis*. Other *Capnocytophaga* species showed ≥6 base mismatches, allowing species-level discrimination. Based on the clinical improvement of the surgical site and molecular diagnostic results, antibiotic therapy was transitioned to oral amoxicillin/clavulanate and discontinued 10 weeks after surgery. Postoperatively, the affected limb was immobilized with a thumb spica cast for four weeks. The duration of antimicrobial therapy was determined in consultation with the infection control team at our institution, considering the patient’s clinical response. No recurrence of infection or pathological fracture was observed during the follow-up.

## Discussion

This report describes two cases of cat bite-associated infections of the upper extremity that required surgical treatment. In both cases, the initial outpatient management and clinical assessment failed to prevent disease progression, ultimately necessitating surgical intervention for adequate source control. Pathogen identification was achieved only after surgical treatment in both cases. In Case 2, conventional culture methods were insufficient, requiring genetic testing to identify the causative organism. These cases highlight the importance of timely surgical intervention and appropriate pathogen identification. Histopathological confirmation was not performed, which represents a limitation of this report. However, the combination of radiological, intraoperative, microbiological, and clinical findings provided converging evidence supporting the diagnosis.

Cat bites account for approximately 20%-30% of all animal bite injuries [1]. Cats possess sharp, elongated teeth that can inflict deep puncture wounds, allowing oral microorganisms to inoculate deep tissues, bones, and joints, leading to deep-seated infections [6]. Therefore, cat bites often present with small wound sites, which may be underestimated during the initial clinical assessment. A previous study reported that 20%-80% of cat bite wounds may become infected [2]. Clinical infection following cat bites is primarily characterized by the acute onset of erythema, swelling, and severe pain within 24 hours after the initial injury in 70% of patients and within 48 hours in nearly 90% of cases [7]. The oral microbiota of cats is diverse, and identifying the causative pathogen is a key factor in determining an appropriate treatment strategy for cat bite-associated infections [8].

*Pasteurella* species are part of the natural oral flora of domestic cats, with up to a 90% carriage rate [9]. *Pasteurella multocida*, a Gram-negative, nonmotile, non-spore-forming, pleomorphic coccobacillus, is the most frequently isolated pathogen in cat bites [4]. Infection with *Pasteurella multocida* typically presents as rapidly spreading cellulitis that develops within 24 hours of a bite [4]. If the treatment is inadequate, the infection may spread to deeper tissues [10]. The characteristics of this temporal progression and development of the infection were similar to those observed in Case 1.

*Capnocytophaga* species are capnophilic, facultatively anaerobic, Gram-negative bacilli that commonly inhabit the oral cavity of humans and domestic animals [11]. In addition to *Pasteurella* species, *Capnocytophaga* species are increasingly recognized as clinically relevant pathogens in cat bite-associated infections [5]. These bacteria can cause a wide spectrum of disease severities, including localized infections and severe systemic involvement [5]. Previous studies have indicated that splenectomy, alcohol abuse, insulin-dependent diabetes mellitus, and immunosuppression are risk factors for progression to severe infection [12,13]. However, caution is warranted because infections have also been reported in immunocompetent hosts [5]. Consistent with these reports, Case 2 was immunocompetent and lacked typical risk factors for severe infection but developed osteomyelitis of the first metacarpal bone. Previous reports have shown that conventional cultures may yield negative results, potentially leading to diagnostic difficulties, whereas genetic testing allows for pathogen identification [5]. In our series, PCR sequencing was performed in Case 2 because conventional cultures were negative despite radiological and intraoperative findings strongly suggestive of osteomyelitis. Although broad-range 16S rRNA PCR identified *Capnocytophaga felis* with 100% sequence identity, the possibility of polymicrobial infection cannot be completely excluded, as molecular techniques may preferentially amplify dominant bacterial DNA. However, no additional sequences were detected, and the clinical course, together with targeted antimicrobial response, supports *Capnocytophaga felis* as the most likely causative organism. Accurate pathogen identification was considered essential to guide targeted antimicrobial therapy in this deep musculoskeletal infection. *Capnocytophaga felis* was first identified as a novel bacterial species in 2020 [14]. Although human infections caused by *Capnocytophaga felis* have rarely been reported, to our knowledge, its progression to osteomyelitis has not been described in the literature to date.

The two cases presented in this report shared the common feature of severe upper extremity infections following cat bites that ultimately required surgical treatment; however, clear differences were observed in their clinical courses and inflammatory findings. Case 1 represented a typical, rapidly progressive infection characterized by evident inflammatory responses and abscess formation. In contrast, Case 2 showed transient clinical improvement after the initial treatment but subsequently followed an indolent and atypical course with minimal inflammatory response, ultimately progressing to osteomyelitis. These contrasting cases demonstrate that cat bite-associated infections do not follow a uniform clinical pattern and may exhibit diverse courses depending on the causative pathogen. Particularly, even when the initial clinical findings appear mild, clinicians should be aware of the potential for occult progression to deep musculoskeletal infections, underscoring the importance of careful reassessment based on clinical course and imaging findings.

Appropriate antimicrobial therapy for cat bite-associated infections should target feline oral flora, including *Pasteurella* and *Capnocytophaga* species. Beta-lactam antibiotics combined with beta-lactamase inhibitors, such as ampicillin/sulbactam or amoxicillin/clavulanate, are recommended [4]. In Case 1, initial treatment with cephalexin may not have provided adequate coverage for *Pasteurella multocida*, possibly contributing to disease progression. Even when appropriate antimicrobial therapy is administered, antibiotic treatment alone may be insufficient after the development of deep musculoskeletal infection. Surgical debridement is essential not only for definitive source control but also for achieving an accurate diagnosis. In both cases, pathogen identification was accomplished only through surgical intervention. These findings underscore the importance of combining timely surgical intervention with appropriate antimicrobial therapies. Furthermore, in cases with atypical clinical courses or culture-negative deep infections, genetic testing may contribute to accurate diagnosis and optimal treatment planning.

## Conclusions

In this study, we report two cases of cat bite-associated infections of the upper extremity that required surgical treatment. Although upper extremity infections after cat bites are often underestimated because of mild initial clinical findings, they carry a significant risk of progressing to deep musculoskeletal infections. The differences in the clinical course and diagnostic effort required between the two cases underscore the diversity of the feline oral microbiota involved in cat bite-associated infections. Timely surgical interventions and accurate pathogen identification are essential for definitive source control and appropriate treatment planning.
